# The impact of nutritional immunity on Group B streptococcal pathogenesis during wound infection

**DOI:** 10.1128/mbio.00304-23

**Published:** 2023-06-26

**Authors:** Madeline S. Akbari, Rebecca A. Keogh, Jana N. Radin, Yamil Sanchez-Rosario, Michael D. L. Johnson, Alexander R. Horswill, Thomas E. Kehl-Fie, Lindsey R. Burcham, Kelly S. Doran

**Affiliations:** 1 Department of Immunology and Microbiology, University of Colorado School of Medicine, Anschutz Medical Campus, Aurora, Colorado, USA; 2 Department of Microbiology, University of Illinois at Urbana-Champaign, Urbana, Illinois, USA; 3 Department of Immunobiology, University of Arizona College of Medicine—Tucson, Tucson, Arizona, USA; 4 Valley Fever Center for Excellence, University of Arizona College of Medicine—Tucson, Tucson, Arizona, USA; 5 BIO5 Institute, University of Arizona College of Medicine—Tucson, Tucson, Arizona, USA; 6 Asthma and Airway Disease Research Center, University of Arizona College of Medicine—Tucson, Tucson, Arizona, USA; 7 Department of Veterans Affairs, VA Eastern Colorado Health Care System, Aurora, Colorado, USA; 8 Carl R. Woese Institute for Genomic Biology, University of Illinois at Urbana-Champaign, Urbana, Illinois, USA; University of Illinois Chicago, Chicago, Illinois, USA

**Keywords:** diabetes, soft tissue infection, Group B streptococcus, innate immunity, ABC transporters, neutrophils

## Abstract

Group B *Streptococcus* (GBS) is a Gram-positive pathobiont that can cause adverse health outcomes in neonates and vulnerable adult populations. GBS is one of the most frequently isolated bacteria from diabetic (Db) wound infections but is rarely found in the non-diabetic (nDb) wound environment. Previously, RNA sequencing of wound tissue from Db wound infections in *lepr^d^*^b^ diabetic mice showed increased expression of neutrophil factors, and genes involved in GBS metal transport such as the zinc (Zn), manganese (Mn), and putative nickel (Ni) import systems. Here, we develop a Streptozotocin-induced diabetic wound model to evaluate the pathogenesis of two invasive strains of GBS, serotypes Ia and V. We observe an increase in metal chelators such as calprotectin (CP) and lipocalin-2 during diabetic wound infections compared to nDb. We find that CP limits GBS survival in wounds of non-diabetic mice but does not impact survival in diabetic wounds. Additionally, we utilize GBS metal transporter mutants and determine that the Zn, Mn, and putative Ni transporters in GBS are dispensable in diabetic wound infection but contributed to bacterial persistence in non-diabetic animals. Collectively, these data suggest that in non-diabetic mice, functional nutritional immunity mediated by CP is effective at mitigating GBS infection, whereas in diabetic mice, the presence of CP is not sufficient to control GBS wound persistence.

**IMPORTANCE:**

Diabetic wound infections are difficult to treat and often become chronic due to an impaired immune response as well as the presence of bacterial species that establish persistent infections. Group B *Streptococcus* (GBS) is one of the most frequently isolated bacterial species in diabetic wound infections and, as a result, is one of the leading causes of death from skin and subcutaneous infection. However, GBS is notoriously absent in non-diabetic wounds, and little is known about why this species thrives in diabetic infection. The work herein investigates how alterations in diabetic host immunity may contribute to GBS success during diabetic wound infection.

## OBSERVATION

### Development of Stz-induced diabetic GBS wound model

**Fig 1:**
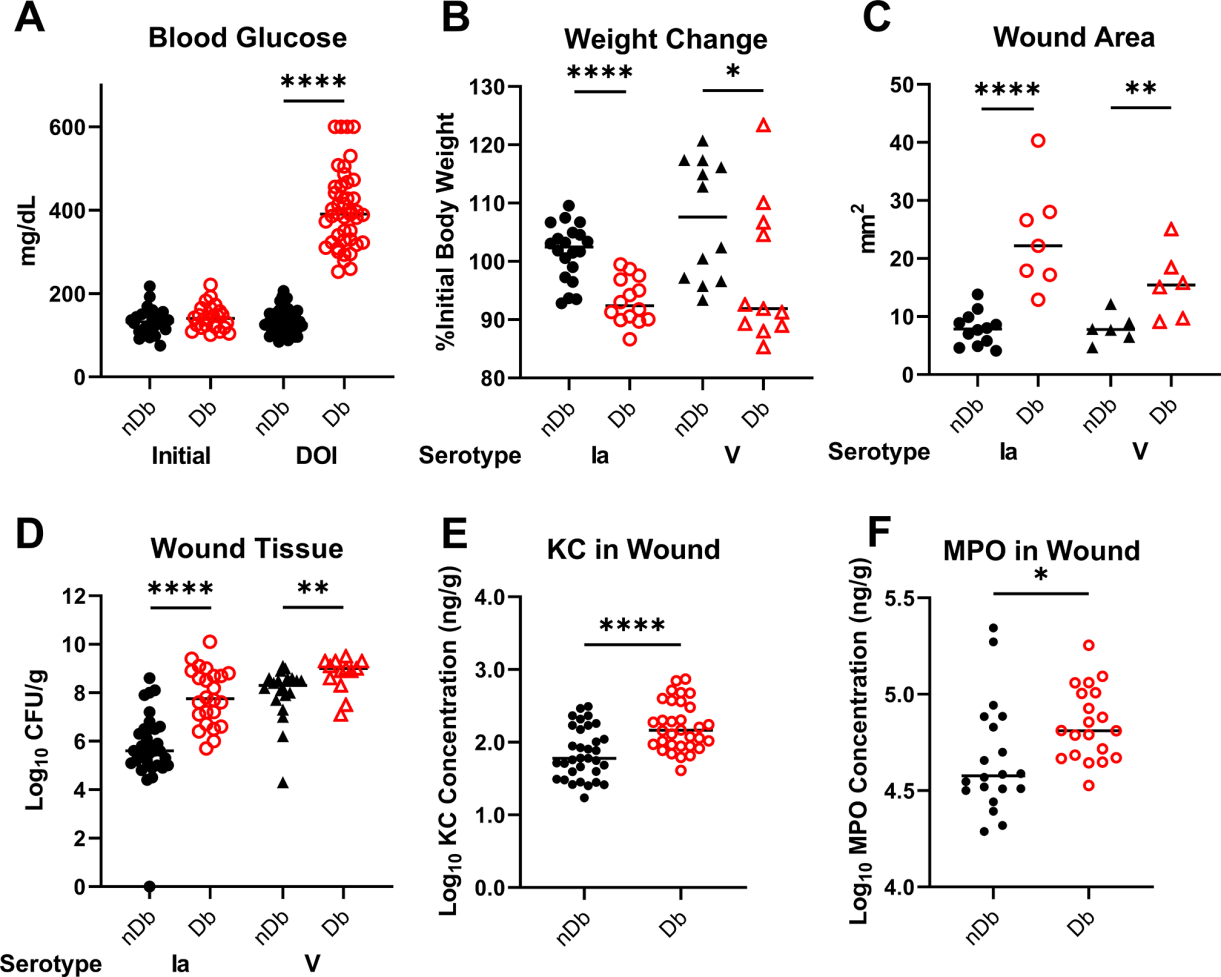
Development of the Stz-induced Db wound model with GBS infection. (A) Non-fasting blood glucose measurements on first day of Stz injections and on DOI. (B) Percent initial body weight calculated from mice weight on DOI and day of sacrifice. (C) Wound area quantified using ImageJ on day of sacrifice. (D) CFU recovered from nDb and Db wound homogenate after GBS infection normalized to tissue weight. (E and F) ELISAs on wound tissue homogenate from day of sacrifice. All concentrations were normalized to tissue weight. Significance determined by (A, E, F) unpaired *t*-test or (B–D) Mann-Whitney *U* test; *P < 0.05, **P < 0.01, and ****P < 0.0001. Db, diabetic; DOI, day of infection; ELISAs, enzyme-linked immunosorbent assays; GBS, Group B *Streptococcus*; nDb, non-diabetic; Stz, Streptozotocin.

We developed a murine model of Group B *Streptococcus* (GBS) diabetic (Db) wound infection using multiple low-dose injections of Streptozotocin (Stz) (Fig. 1A) (1). Mice were wounded with a 6-mm biopsy punch and infected with GBS strains A909 (serotype Ia) or CJB111 (serotype V) (2). These strains represent the two most common serotypes to cause invasive disease in non-pregnant adults and are commonly isolated from diabetic wounds (2 - 5). Diabetic (Db) mice lost more weight, had larger wounds, and had increased bacterial burden in wound tissue regardless of GBS strain compared to non-diabetic (nDb) mice (Fig. 1B through D). We saw no significant differences in colony forming units (CFU) recovered from male and female mice (Fig. S1A). We observed that neutrophil chemoattractant KC (keratinocyte-derived cytokine, CXCL1) and myeloperoxidase (MPO) were at significantly higher concentrations in wound tissue from infected Db mice compared to nDb (Fig. 1E and F). These results parallel the *lepr^d^*^b^ model of GBS Db wound infection where GBS contributes to inflammation by stimulating neutrophil-mediated immune responses (2).

### Loss of calprotectin does not increase GBS growth in diabetic wounds

**Fig 2:**
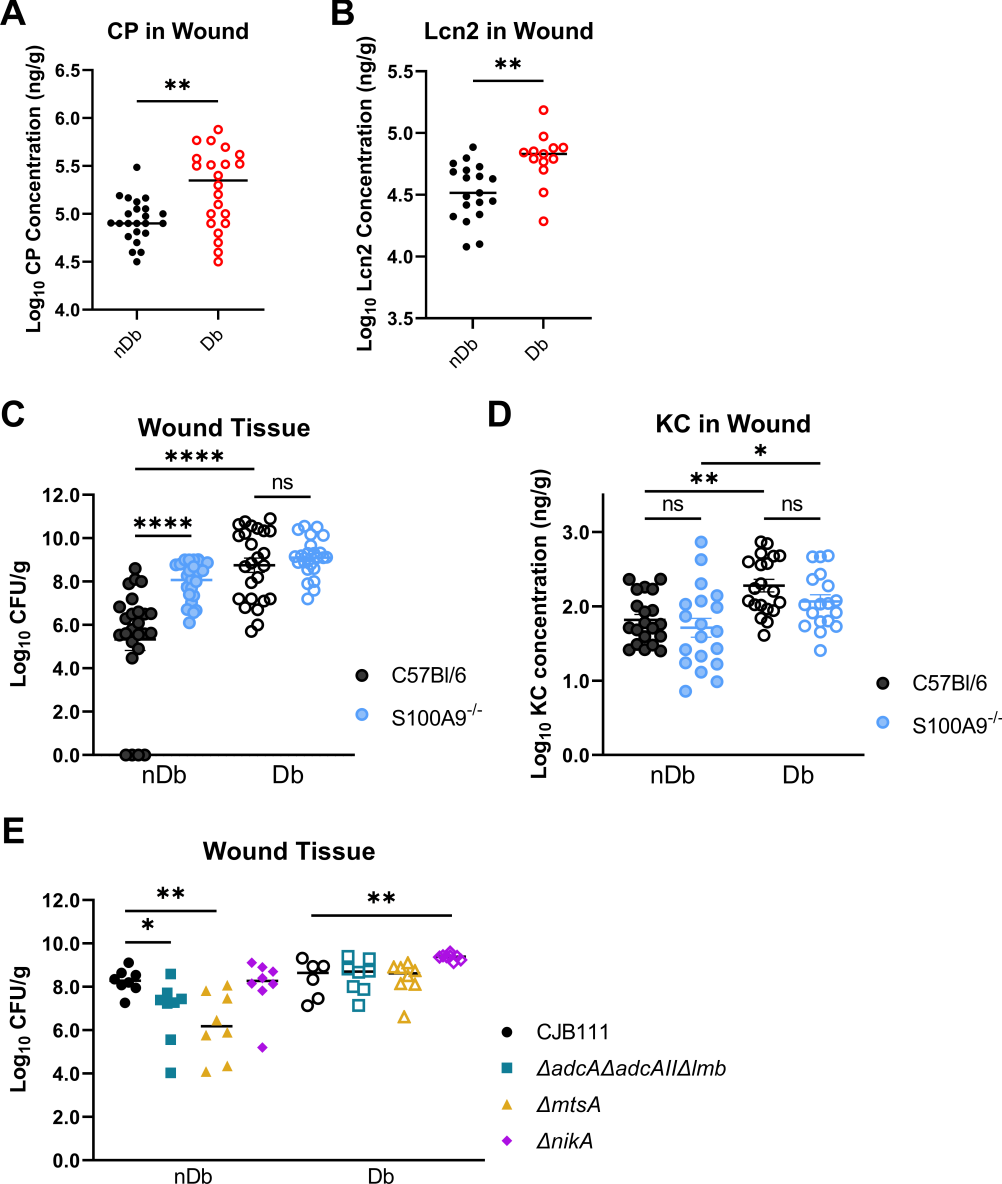
Metal homeostasis during GBS Db wound infection. (A and B) ELISAs on wound tissue homogenate from day of sacrifice. All concentrations were normalized to tissue weight. (C) CFU recovered from nDb and Db wound homogenate infected with GBS from C57Bl/6 and *S100A9^−/−^* mice. (D) ELISAs on wound tissue homogenate from day of sacrifice. All concentrations were normalized to tissue weight. (E) CFU recovered from nDb and Db wound homogenate infected with wild-type (WT), Zn (*adcAadcAIIlmb*), Mn (*mtsA*), or putative Ni (*nikA*) transporter mutant. Significance determined by (A and B) unpaired *t*-test, (C and D) one-way ANOVA with Šídák’s multiple comparisons test, or (E) Mann-Whitney *U* test; *P < 0.05, **P < 0.01, and ****P < 0.0001. ANOVA, analysis of variance; Db, diabetic; nDb, non-diabetic; ELISAs, enzyme-linked immunosorbent assays; GBS, Group B *Streptococcus*; Mn, manganese; Zn, zinc.

In previous studies, dual-RNA sequencing on nDb and Db infected wound tissues revealed that factors involved in host and bacterial metal homeostasis were induced during GBS infection (2). Specifically, host genes involved in metal sequestration including both subunits of CP (*S100a8* and *S100a9*) and lipocalin-2 (LCN2) were upregulated in Db infection in comparison to nDb. CP is capable of chelating bioavailable zinc (Zn), manganese (Mn), iron, and nickel (Ni), while LCN2 sequesters iron by binding bacterial ferric siderophores (6 - 9). Both proteins are involved in the host defense mechanism termed nutritional immunity where pathogens are starved of important nutrients (10). In our Stz-induced diabetic model we confirmed that CP and LCN2 abundances were increased during infection in wound tissue from Db mice compared to nDb mice (Fig. 2A and B). We observed that wounding alone in the absence of infection increased CP and LCN2 concentrations compared to non-wounded skin tissue taken from the same mice, but these were lower than concentrations measured from infected wounds and there were no differences between Db and nDb mice (Fig. S1B and C).

To elucidate the role of CP-mediated metal sequestration during GBS wound infection we utilized a CP knock-out mouse strain (*S100A9^−/−^)* in our model. In nDb wounds, there was a significant increase in recovered CFU from *S100A9^−/−^* mice compared to the wild-type mouse strain (C57Bl/6J); however, there was no difference between Db WT or *S100A9^−/−^* mice (Fig. 2C). This suggests that the contribution of CP-mediated nutritional immunity to limiting GBS survival is more effective in nDb mice. Of note, CP can be pro-inflammatory via binding to Toll-like receptor 4 (TLR4) which could impact inflammation, particularly neutrophil recruitment to the site of infection (11). However, we did not observe any difference in KC abundance in wound tissue between WT and *S100A9^−/−^* mice regardless of diabetic status (Fig. 2D).

### GBS metal transport systems are dispensable during diabetic infection

As we previously observed, bacterial genes for the substrate binding proteins of Zn (*adcA*, *adcAII*, and *lmb*), Mn (*mtsA*), and a putative Ni (*nikA*) transport systems in GBS were all 10- to 20-fold upregulated during nDb and Db infection compared to GBS grown in culture (input) (2); however, these metal transport systems were less induced during Db infection when compared to nDb despite the increase in CP. We hypothesized metal homeostasis would be important for GBS survival in both environments. One of the most highly induced systems was a putative Ni ABC-transport system in GBS consisting of five genes, *nikABCDE* (Fig. S2A), homologous to the nickel system in *Escherichia coli*, but uncharacterized in GBS (Fig. S2B) (12). We constructed a mutant in the substrate binding protein (NikA) and performed inductively coupled plasma optical emission spectrometry (ICP-OES) on WT and *nikA* pellets to initially characterize the NikABCDE transporter by measuring total cell associated and intracellular ion concentrations (Fig. S2C). Results show that total and intracellular copper (Cu) were significantly lower for *nikA* compared to WT. The *nikA* mutant, along with a previously described Zn substrate binding mutant (*adcAadcAIIlmb*) (13), and Mn substrate binding mutant (*mtsA*) (14) were tested for their ability to survive in wound infection. In the Db environment, none of the GBS mutants exhibited attenuated survival compared to the WT strain, whereas the *mtsA* and *adcAadcAIIlmb* mutants had decreased bacterial persistence in nDb wounds (Fig. 2E). This was also observed in the *lepr^d^*^b^ Db wound model for *adcAadcAIIlmb* (Fig. S3). These results suggest that GBS is less starved for metals in the Db wound and therefore has less requirement for metal import systems.

### Conclusions

Here, we utilize an Stz-induced model of diabetes and find that Db mice have increased disease severity, markers of inflammation, and GBS burden in comparison to nDb mice, supporting previous work on GBS diabetic wound infection in *lepr^db^* mice (2). Using this model, we were able to assess the contribution of host CP to GBS wound persistence.

Nutritional immunity by the host involves sequestering nutrients from invading pathogens, effectively preventing growth (10, 15). CP and LCN2 are neutrophil associated host factors involved in this process with CP being one of the most abundant immune proteins at sites of infection (16). CP is released in neutrophil extracellular traps along with MPO, elastase and other antimicrobial proteins, while LCN2 is secreted in neutrophil granules at sites of inflammation (7, 17). CP has been shown to inhibit the survival and growth of GBS and other pathogens such as *Staphylococcus aureus* and *Clostridium difficile* (18 - 20). Here, we show that CP limits GBS survival in nDb wounds, suggesting that proper nutritional immunity in the nDb mouse may mediate bacterial clearance. CP is also increased in Db wounds and is used as a marker for infection, but its role and contribution to Db infection are unknown (21). We confirm that CP concentrations are increased in Stz-induced Db wounds during GBS infection, but CP is surprisingly dispensable in controlling GBS infection. Thurlow et al. previously demonstrated that neutrophils from Db mice had reduced phagocytic activity and respiratory burst during *S. aureus* infection compared to nDb mice (22). If the same is true during GBS infection, a lack of respiratory burst may indicate that while CP is present, it is not released from the neutrophil to mitigate bacterial clearance. We further speculate that a diminished respiratory burst may limit the need for Mn-dependent superoxide dismutase in GBS and therefore, limit the need for Mn transport in this niche (23). CP is also known to bind the receptor of advanced glycation end products, which is increased in Db individuals and could further disrupt CP-mediated metal chelation in the Db wound (24). The surface of some pathogens like *Listeria monocytogenes* has also been shown to interact with CP and can affect pathogen binding and uptake in epithelial cells but this remains to be investigated in the context of the Db wound (25).

Furthermore, the loss of metal transporters did not significantly reduce GBS recovery from Db wounds but did reduce Zn and Mn substrate binding mutant recovery from nDb wounds. We speculate that the bacterial cellular demand for metals is less in Db wounds than nDb wounds and/or metals are more freely available to the bacteria in the Db wound due to the altered immune response. Interestingly, we recovered similar or higher bacterial loads from wounds infected with the Δ*nikA* mutant compared to WT GBS, regardless of diabetic status. Genes in the Nik operon have been shown to be induced in other niches, such as during GBS vaginal colonization and periprosthetic joint infection, and under CP stress (14, 26, 27). Our ICP-OES analysis indicates that NikABCDE may be a promiscuous import system, including Cu import, the impact of which is unknown. Historically, Gram-positive bacteria do not have a need for Cu import systems (28), thus further study on specific metals being transported by the Nik system in various environments will be of interest.

In summary, metal homeostasis at the site of infection is a significant process that is known to impact pathogen survival and it is important to understand in different niches. These observations suggest that metal limitation is disrupted in the Db wound and therefore, we speculate that nutritional immunity is either non-functioning or not sufficient to control infection in the hyper-inflammatory environment. Therapies or drug targets that deplete metals may aid the impaired immune response to infection in Db wounds and help clear pathogens.

## SUPPLEMENTAL MATERIAL

Supplemental figures mbio.00304-23-s0001.docx Figures S1 to S3.

TABLE S1 mbio.00304-23-s0002.xlsx Table of specific strains and primers used.

Supplemental text mbio.00304-23-s0003.docx Methods for all experiments performed.

ASM does not own the copyrights to Supplemental Material that may be linked to, or accessed through, an article. The authors have granted ASM a non-exclusive, world-wide license to publish the Supplemental Material files. Please contact the corresponding author directly for reuse.
