# Supplementary material for: The impact of nutritional immunity on Group B streptococcal pathogenesis during wound infection
Source: mBio. 2023 Jun 26;14(4):e00304-23. doi: 10.1128/mbio.00304-23 (PMC10470527; doi:10.1128/mbio.00304-23)
Supplement: Supplemental figures — Figures S1 to S3. [file mbio.00304-23-s0001.docx]

**Figure S1.** **Further characterization of Stz-induced diabetic wound model.** **A)** CFU recovered from nDb and Db male and female wounds after GBS infection normalized to tissue weight. Significance determined by 2way ANOVA with multiple comparisons test; ***P* < 0.01. **B&C)** ELISAs on skin tissue homogenate taken on day of wounding and wound tissue homogenate from day of sacrifice. All concentrations were normalized to tissue weight. Significance determined by Mann-Whitney *U* test; ***P* < 0.01.


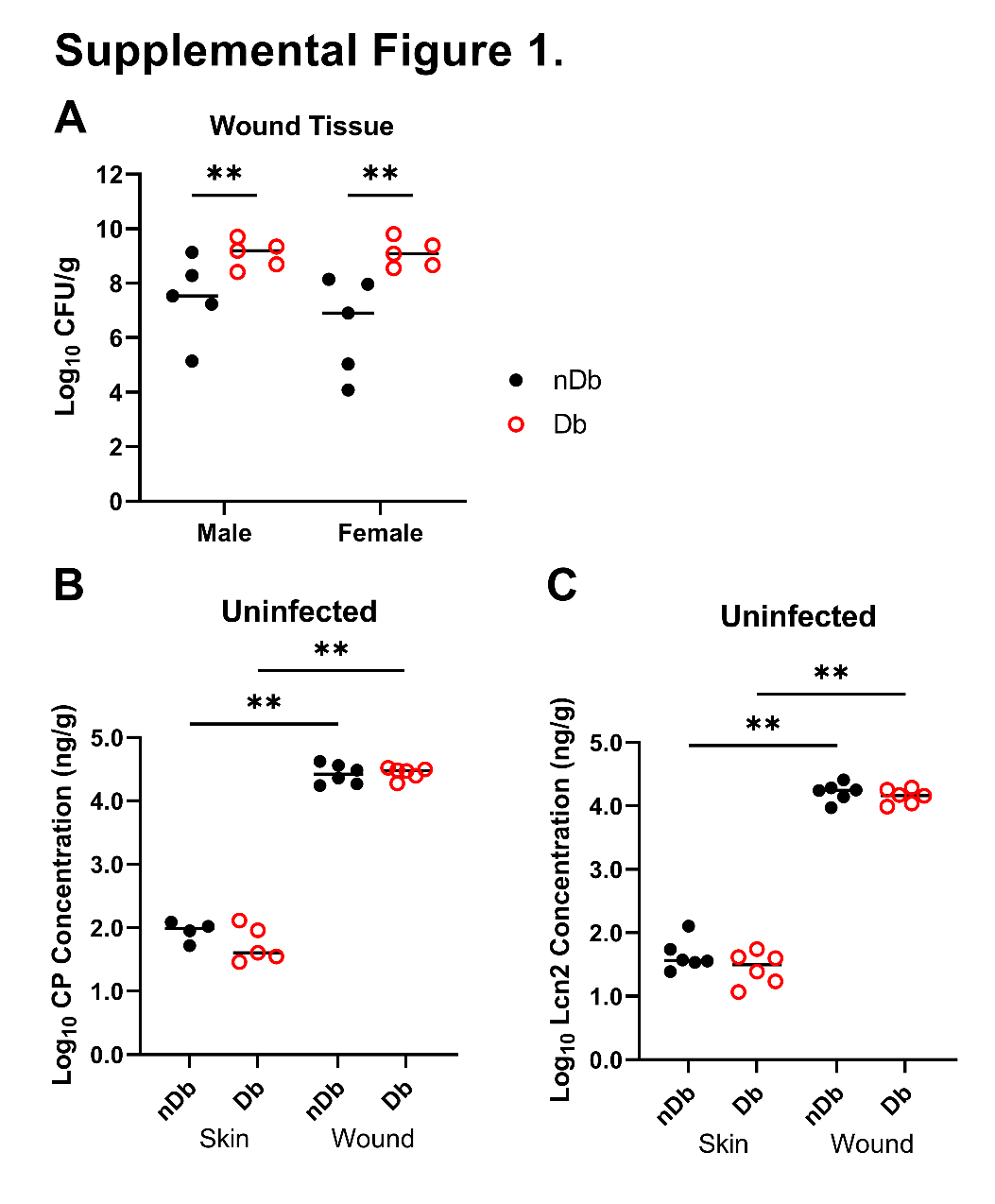


**Figure S2. *nikABCDE* operon identification and characterization. A)** Diagram of *nikABCDE* genes in operon. Genes are in reverse complement direction. **B)** Percent identify and amino acid sequence alignment for the substrate binding protein, NikA, from *E. coli* (top) and GBS (bottom). **C)** ICP-OES on cultures pellets for WT GBS and Δ*nikA* strains. Significance determined by Unpaired t test; **P* < 0.05 and ***P* < 0.01.


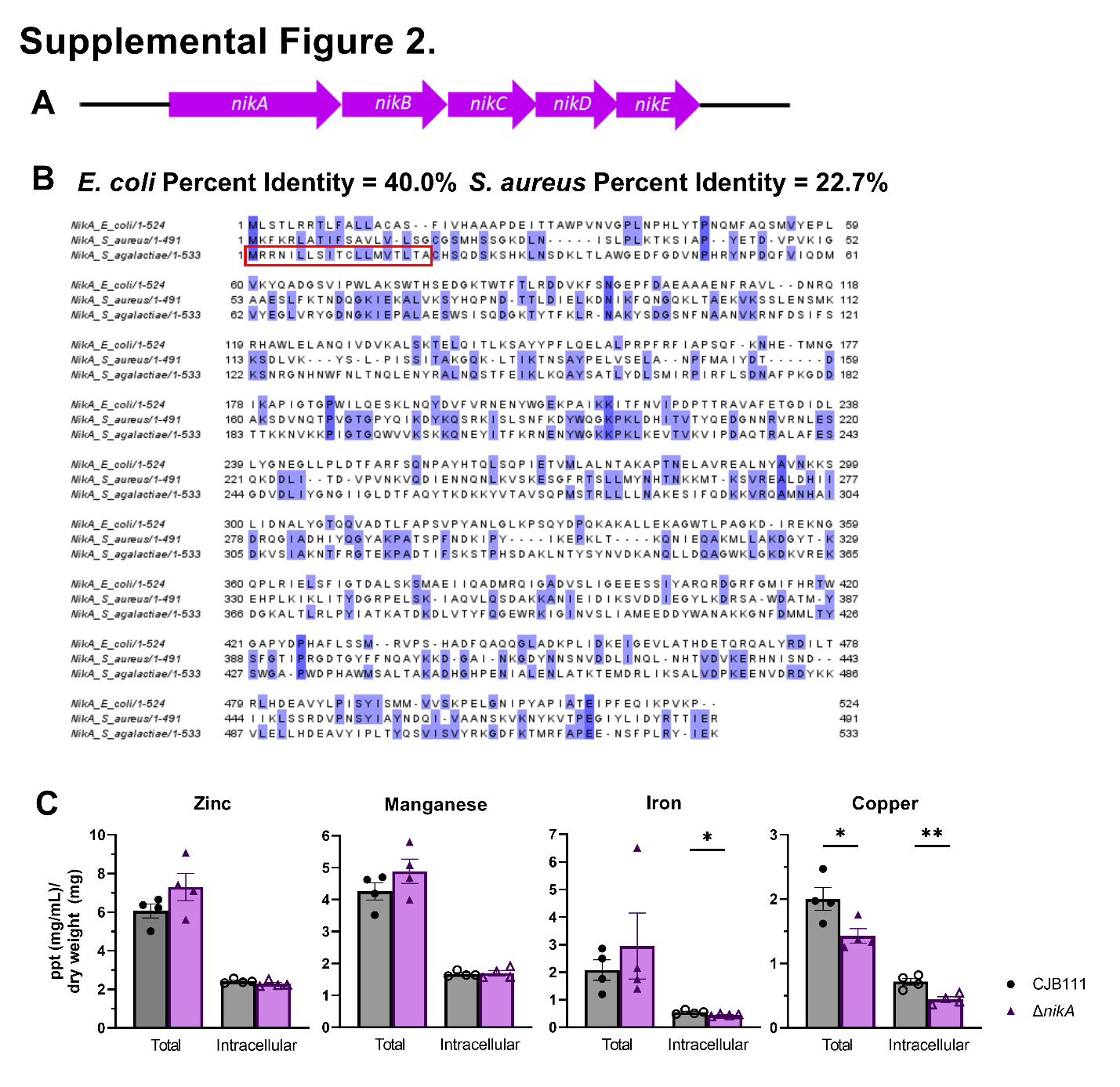


**Figure S3. Zinc transporter mutant in non-diabetic and *lepr^d^*^b^ diabetic wound infection.** CFU recovered from nDb and *lepr^db^* Db wound homogenate infected with WT GBS or Zn (Δ*adcA*Δ*adcAII*Δ*lmb*) transporter mutant. Significance determined by Mann-Whitney *U* test; ***P* < 0.01.


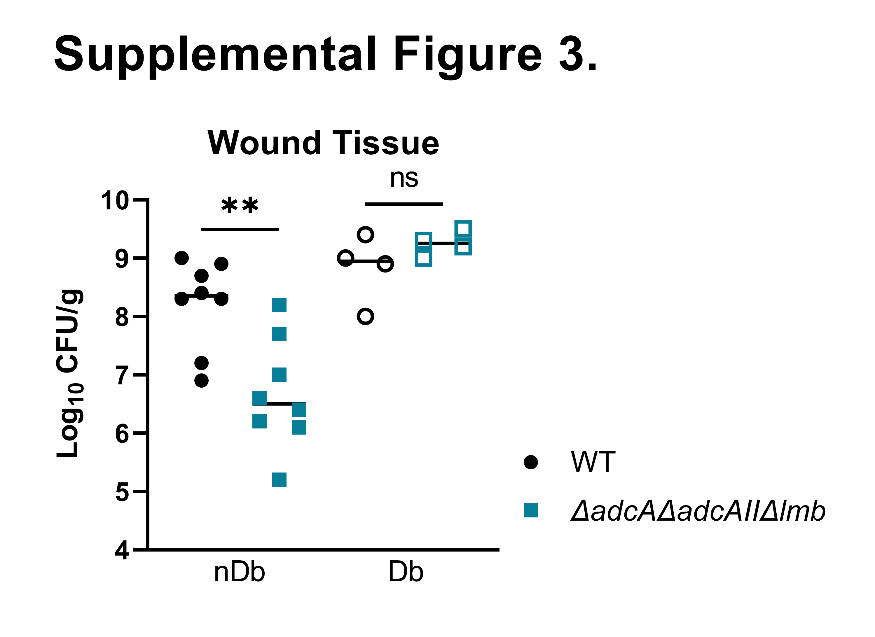


**Table 1. List of strains and primers used in this study.**
