# Supplementary material for: The impact of nutritional immunity on Group B streptococcal pathogenesis during wound infection
Source: mBio. 2023 Jun 26;14(4):e00304-23. doi: 10.1128/mbio.00304-23 (PMC10470527; doi:10.1128/mbio.00304-23)
Supplement: Supplemental text — Methods for all experiments performed. [file mbio.00304-23-s0003.docx]

**Supplemental Methods.**

Methods for all experiments performed and data presented in the manuscript.

***Study Approval.*** Animal experiments were approved by the Institutional Animal Care and Use Committee (IACUC) at the University of Colorado Anschutz Medical Campus protocol #00987 and were performed using accepted veterinary standards. The University of Colorado Anschutz Medical Campus is AAALAC accredited; and its facilities meet and adhere to the standards in the “Guide for the Care and Use of Laboratory Animals”.

***Bacterial Strains and Growth Conditions.*** *Streptococcus agalactiae* (GBS) isolates A909 (serotype Ia), CJB111 (serotype V), zinc transporter mutant strain (Δ*adcA*Δ*adcAII*Δ*lmb*), manganese transporter mutant strain (Δ*mtsA*), and putative nickel transporter mutant strain (Δ*nikA*) were statically cultured in Todd-Hewitt Broth (THB) at 37°C. The mutant strains were generated as previously described (1-3). Briefly, for the *nikA* mutant, genomic 5’ and 3’ regions flanking the *nikA* gene were amplified and fused with a spectinomycin cassette by FailSafe PCR (Lucigen). Fragments and pHY304 vector were digested with restriction enzymes XhoI and XmaI and ligated using a Quick Ligation kit (NEB). The ligation reaction product was transformed into chemically competent *Escherichia coli* MC1061 and selected on LB agar with 500µg/mL erythromycin. pHY304 plasmids were purified from *E. coli* and elecroporated into GBS A909 and CJB111 genetic backgrounds and selected on Todd-Hewitt agar (THA) plates with 100µg/mL spectinomycin. Construct was confirmed by PCR and sequencing. Primers used in the construction of the *nikA* mutant and all strains used are listed in **Table S1**.

***Mice***. All mice were housed in pathogen-free, biosafety level-2 animal facilities in accordance with the Institutional Animal Care and Use Committee of the University of Colorado (protocol #00987). C57BL/6J *S100A9^-/-^* mice for WT GBS challenges were provided by the Kehl-Fie Lab. C57BL/6J mice for WT and mutant GBS challenges were purchased from Jackson Laboratories.

***Murine model of diabetes.*** Female 7-week-old *lepr^db^* mice were used or to develop diabeties, female or male 7-week-old C57Bl/6J mice or female 6-12-week-old *S100A9^-/-^* mice were given multiple low-dose intraperitoneal injections of Streptozocin (Stz). Each dose contained 50-120 mg/kg of Stz (CAS-No: 18883-66-4) dissolved immediated before injection in 100uL 50 mM sodium citrate buffer (pH 4.5). Injections were given every 2-3 days until mice developed diabetes (average of 5 injections total). Mice with glucose concentrations exceeding 250 mg/dL were considered diabetic. Blood glucose was measured using a glucometer with blood obtained by tail prick. Body weight and blood glucose were recorded on day of first Stz injection, on day of infection, and on day of sacrifice.

***Murine model of GBS wound infection.*** We utilized a mouse model of GBS wound infection as previously described (4). Briefly, while under anesthesia, the mice underwent a wounding procedure where a 6 mm biopsy punch of their shaved back is removed and 1 x 10^7^ CFU GBS (A909), 1x 10^6^ CFU GBS (CJB111), or 10µL of PBS (Fisher BioReagents Code: BP2944) was added before wrapping the mice in adhesive Tegaderm^TM^. The biopsy punch of skin tissue was saved when indicated, homogenized in sterile PBS, and plated on CHROMagar to confirm GBS absense. The adhesive was removed 72 h post-wounding and then mice were sacrificed 24 h later. Wound tissue was removed and homogenized in sterile PBS and plated on CHROMagar to quantify GBS CFU burden.

***ELISAs on wound homogenates.*** Proteins in homogenized tissues were quantified using R&D system ELISA kits (catalog # DY8596-05, DY453, DY3667, MLCN20). Protein detected was normalized to tissue weight and reported as protein (ng) per mg of tissue.

***NikA pairwise alignment.*** The amino acid sequences for *Escherichia coli* NikA (Uniprot P33590, Q2M7D9), *Staphylococcus aureus* NikA (Uniprot A0A0H2UKY7) and *Streptococcus agalactiae* NikA (Uniprot A0A7Z7QT79) were aligned and percent identity calculated using Jalview version 2.11.2.5 for Windows (5). The predicted signal peptidase II cleavage site for *S. agalactiae* NikA is boxed in red.

***ICP-OES analysis.*** Overnight cultures of GBS CJB111 WT and Δ*nikA* strains were back diluted into fresh THB and then grown to an OD_600_ of 0.4. 10mL of culture was pelleted for each sample. Cell pellets were then rinsed in 1 mL nuclease-free H_2_O and pelleted again. Cell pellets used to measure intracellular metal concentration were also washed with 1 mL of 0.5M EDTA and pelleted again. All samples were desiccated at 65°C for 1 h. For inductively coupled plasma optical emission spectrometry (ICP-OES), bacterial pellets were digested at 95°C for 15 minutes in 143 μL nitric acid, then resuspended to 5 mL with Milli-Q water. A Thermo Scientific iCAP PRO XDUO ICP-OES was used for the analysis. Zn, Mn, Fe, and Cu concentrations in parts per thousand (mg/mL) were determined by plotting samples against a standard curve and were normalized across bacterial strains by cell dry weight. Ni concentrations were below the limit of detection. The data are represented as the mean Zn, Mn, Fe, and Cu concentration from four independent cultures of WT or Δ*nikA* mutant strains.

***Statistical analysis.*** Statistical analysis was performed using Prism version 9.4.0 (673) for Windows (GraphPad Software, San Diego, CA, USA) as described in figure legends.

1. Burcham LR, Le Breton Y, Radin JN, Spencer BL, Deng L, Hiron A, Ransom MR, Mendonça JDC, Belew AT, El-Sayed NM, McIver KS, Kehl-Fie TE, Doran KS. Identification of Zinc-Dependent Mechanisms Used by Group B Streptococcus To Overcome Calprotectin-Mediated Stress. mBio. 2020 Nov 10;11(6):e02302-20. doi: 10.1128/mBio.02302-20. PMID: 33173000; PMCID: PMC7667036.
2. Burcham LR, Akbari MS, Alhajjar N, Keogh RA, Radin JN, Kehl-Fie TE, Belew AT, El-Sayed NM, McIver KS, Doran KS. Genomic Analyses Identify Manganese Homeostasis as a Driver of Group B Streptococcal Vaginal Colonization. mBio. 2022 Jun 28;13(3):e0098522. doi: 10.1128/mbio.00985-22. Epub 2022 Jun 6. PMID: 35658538; PMCID: PMC9239048.
3. Spencer BL, Tak U, Mendonça JC, Nagao PE, Niederweis M, Doran KS. A type VII secretion system in Group B Streptococcus mediates cytotoxicity and virulence. PLoS Pathog. 2021 Dec 6;17(12):e1010121. doi: 10.1371/journal.ppat.1010121. PMID: 34871327; PMCID: PMC8675928.
4. Keogh RA, Haeberle AL, Langouët-Astrié CJ, Kavanaugh JS, Schmidt EP, Moore GD, Horswill AR, Doran KS. Group B *Streptococcus* adaptation promotes survival in a hyperinflammatory diabetic wound environment. Sci Adv. 2022 Nov 11;8(45):eadd3221. doi: 10.1126/sciadv.add3221. Epub 2022 Nov 11. PMID: 36367946; PMCID: PMC9651866.
5. Waterhouse AM, Procter JB, Martin DM, Clamp M, Barton GJ. Jalview Version 2--a multiple sequence alignment editor and analysis workbench. Bioinformatics. 2009 May 1;25(9):1189-91. doi: 10.1093/bioinformatics/btp033. Epub 2009 Jan 16. PMID: 19151095; PMCID: PMC2672624.
